# Dietary Inflammatory Index and Head and Neck Cancer: A Multicenter Case-Control Study in Iran

**DOI:** 10.34172/jrhs.2024.159

**Published:** 2024-07-31

**Authors:** Saba Narmcheshm, Monireh Sadat Seyyedsalehi, Bahareh Sasanfar, Hamideh Rashidian, Maryam Hadji, Elham Mohebbi, Ahmad Naghibzadeh-Tahami, Paolo Boffetta, Fatemeh Toorang, Kazem Zendehdel

**Affiliations:** ^1^Department of Community Nutrition, School of Nutritional Sciences and Dietetics, Tehran University of Medical Sciences, Tehran, Iran; ^2^Cancer Research Center, Cancer Institute, Tehran University of Medical Sciences, Tehran, Iran; ^3^Department of Medical and Surgical Sciences, University of Bologna, Italy; ^4^Nutrition and Food Security Research Center, Shahid Sadoughi University of Medical Sciences, Yazd, Iran; ^5^Department of Nutrition, School of Public Health, Shahid Sadoughi University of Medical Sciences, Yazd, Iran; ^6^Health Sciences Unit, Faculty of Social Sciences, Tampere University, Tampere, Finland; ^7^School of Medicine, Department of Oncology, Georgetown University, Washington, DC, USA; ^8^Modeling in Health Research Center, Institute for Futures Studies in Health, Kerman University of Medical Sciences, Kerman, Iran; ^9^Health Foresight and Innovation Research Center, Institute for Futures Studies in Health, Kerman University of Medical Sciences, Kerman, Iran; ^10^Stony Brook Cancer Center, Stony Brook University, Stony Brook, NY, USA; ^11^Department of Family, Population and Preventive Medicine, Renaissance School of Medicine, Stony Brook University, Stony Brook, NY, USA; ^12^Cancer Biology Research Center, Cancer Institute, Tehran University of Medical Sciences, Tehran, Iran

**Keywords:** Dietary inflammatory index, Diet, Head and neck neoplasms, Case-control studies

## Abstract

**Background:** The inflammatory potential of diet may affect carcinogenesis. This study aimed to determine the association between dietary inflammatory index (DII) and the risk of head and neck cancer (HNC), as well as the interaction between DII and cigarette smoking in HNC development within the Iranian population.

**Study Design:** This is a case-control study.

**Methods:** In this multicenter case-control study, participants’ dietary intake was assessed using a validated 130-item food frequency questionnaire, from which DII was computed. The study recruited 876 new cases from referral hospitals across 10 provinces and 3409 healthy controls who were frequency-matched based on age, gender, and residential place. Logistic regression was used to obtain odds ratios (ORs) for HNC across tertiles of DII, which were adjusted for confounding variables.

**Results:** A higher pro-inflammatory diet was associated with an increased risk of all HNC (OR T3 vs. T1 [95% CI]: 1.31 [1.06, 1.62]; *P*-trend=0.013). There was a significant association between lip and oral cavity cancers and DII (OR T3 vs. T1 [95% CI]: 1.56 [1.16, 1.66]; *P*-trend=0.004). Furthermore, an inflammatory diet was associated with an increased risk of pharynx cancer (OR T3 vs. T1 [95% CI]: 2.08 [1.14, 3.79]; *P*-trend=0.02). Additionally, no significant association was observed between DII and larynx cancer, while an interaction was found between DII and tobacco use on the risk of HNC (OR T3 vs. T1 [95% CI]: 2.52 [1.78, 3.57]; *P*-interaction=0.03).

**Conclusion:** DII was positively associated with HNC risk. There was a significant association between DII and the risk of lip, oral cavity, and pharynx cancers. Additionally, there was an interaction between tobacco use and DII in determining the risk of HNC.

## Background

 The global burden of diseasse study in 2017 reported that 5.3 % of all cancers are related to head and neck cancer (HNC).^1^ HNC includes cancers of the oral cavity, oropharynx, nasopharynx, hypopharynx, and larynx. The HNC’s prevalence has recently increased in Iran, with the age-standardized rate rising from 4.8 per 100 000 in 2003 to 8.5 in 2008.^2^ Patients with HNC have different complications including, breathing and eating difficulties, and cancer treatment imposes a significant financial burden on patients and their families.^3^ Therefore, prevention is the best strategy for all people, and identifying the risk factors is vital.

 Tobacco (both cigarette and waterpipe) and alcohol consumption are major risk factors for HNC.^4^ Other important risk factors include asbestos exposure, human papillomavirus infection, and opium use.^5,6^ Evidence shows the protective effect of fruits and vegetables against HNC.^4^ However, the recent comprehensive review of nutritional factors and cancer risk by the World Cancer Research Fund and the American Cancer Institute concluded that the data on the association between several dietary components with HNC is not convincing.^5^

 Chronic inflammation has been recognized as one of the possible mechanisms of carcinogenesis, including HNC.^7^ Dietary components have been shown to have anti-inflammatory and pro-inflammatory effects.^8^ Dietary components such as phenols, antioxidant vitamins, and other nutrients have been associated with the development of several cancers. Some studies have investigated the association between one or several foods or nutrients and cancer risk separately; however, dietary components are consumed together and have synergic or antagonistic interactions. To deal with this, the Dietary Inflammatory Index (DII) was proposed to compute the inflammatory effects of the overall diet.^9^ Several studies have investigated the association between DII and cancers such as colorectal, breast, prostate, and endometrium.^10,11^ Few studies have been conducted on the association between DII and aerodigestive tract cancer,^12,13^ and most data come from non-Asian countries with different dietary habits.^14-16^ As such, studies on the association between DII and the risk of HNC, especially in southwestern Asia, are rare. The present study used a large case-control study among the Iranian population to determine the association between DII and the risk of HNC development within the Iranian population.

## Methods

###  Participants and study design

 The Iranian Study of Opium and Cancer (IROPICAN) is a multicenter case-control study conducted between 2018 to 2020 in referral hospitals affiliated with Universities of Medical Sciences in 10 provinces of Iran, including Sistan and Baluchestan, Kerman, Bushehr, Hormozgan, Khorasan Razavi, Golestan, Mazandaran, Kermanshah, and Tehran.^17^ We selected cases and controls from the IROPICAN study. There were 876 pathologically confirmed HNC patients who received a cancer diagnosis in less than one year and with no history of any cancers. HNCs in this study were the ICD-O-3 codes of HNC, including the oral, larynx, and pharyngeal cancers (i.e., C00-C09, C11, C12, C14, C31, and C32).

 We included 3409 controls who were frequently matched with cases based on age (in 5-year intervals), gender, and residential place. They were selected from hospital visitors who were without cancer, were not family members or friends of the cancer patients, and were visiting the hospital for reasons other than their health complaints.

 Participants were asked detailed questions about opium use and its history (e.g., age at initiation, duration, frequency, typical amount, and route), tobacco use (e.g., cigarettes, naswar, chopogh, and waterpipe), history of alcohol consumption, demographic and socioeconomic factors (e.g. age, ethnicity, rural/urban status, education, and occupational history), physical activity (using the validated International Physical Activity Questionnaire, IPAQ^18^). Information on various aspects of health (personal and family history of cancer) was also obtained. Socioeconomic status (SES) was determined by combining data on education, income, and ownership of some household appliances using principal component analysis. The SES score determined for the control group was used to classify participants into three groups: high, medium, and low social classes. Physical activity workload (PPWL) was estimated based on the job history of participants using the Finland Job Exposure Matrix (FINJEM) ^19^. Based on the PPWL scores in control groups, participants were divided into three groups: sedentary (zero PPWL-years), moderate (PPWL-years above zero and less than or equal to 4.80), and heavy (PPWL-years above 4.80). Dental health was assessed by considering decayed, missing, and filled teeth, and participants were divided into three groups based on the scores of the control group.

###  Assessment of dietary intake and dietary inflammatory index scoring

 To assess the dietary intake of participants, we used a validated 130-item food frequency questionnaire (FFQ) which was designed for Persian Cohort Study.^20^ Cases and controls were asked to answer the FFQ based on their dietary habits one year before cancer diagnosis and one year before the interview, respectively. Total energy and nutrient intake were calculated using the USDA Food Composition Table.^21^ The DII score was computed based on the method suggested by Shivappa using 38 food parameters, including energy, carbohydrate, fat, protein, fiber, cholesterol, mono-unsaturated fatty acids (MUFA), polyunsaturated fatty acids (PUFA), n-3 fatty acids, n-6 fatty acids, saturated fats (SFAs), trans fat, thiamin, riboflavin, niacin, pyridoxine, folic acid, cobalamin, vitamins A, C, and D, alpha-tocopherol, β-carotene, zinc, selenium, magnesium, iron, caffeine, onion, garlic, black tea, coffee, flavan-3-ol, flavones, flavonols, flavonones, anthocyanidins, and isoflavones.^9^ Then, the residual method was used to calculate energy-adjusted quantities of all these parameters before analyzing the DII score.^22^

###  Statistical analysis

 All statistical analyses were performed by Stata software (Stata 14.1, College Station, Texas 77845 USA). Two-sided *P* values < 0.05 were considered statistically significant. Participants were classified into tertiles of DII scores based on the distribution in the control group. To examine the association between DII and odds of HNC, unconditional logistic regression analysis was used which was controlled for several covariates. The full model was adjusted for energy (kcal/d), age (years), gender (female, male), province (10 provinces), socioeconomic status (low, medium, and high), tobacco use (yes, no), opium use (yes, no), alcohol use (yes, no), dental health (poor, moderate, and good), and physical activity (sedentary, moderate, and high). The trend of odds ratios (ORs) was examined using the median of DII as a continuous variable in the logistic regression models. An interaction term was added to models to analyze the interaction between DII and tobacco or opium use and the risk of HNC. The *P* value for interactions was estimated by the likelihood ratio test between models with and without the interaction term. We recruited 894 HNC patients from the IROPICAN study and 3483 apparently healthy controls whose data has been used in this study. Participants with unusual energy intake (less than 500 and over 4500 kcal/d) were considered outliers (18 subjects out of 894 patients and 74 subjects out of 3484 controls) and were omitted.

## Results

 Totally 876 patients and 3409 healthy controls were recruited for the present study. Almost a quarter of the participants in both groups were female (Table 1). Tobacco smoking and opium use were more common among the cases. Regular alcohol use was scarce in both groups. The physical activity status of the participants in both groups was almost similar. Energy and dietary intakes did not significantly differ between patients and controls (Table 2). As observed, the intake of energy, carbohydrate, and trans fatty acids increased from tertile 1 to tertile 3 of DII. However, the intake of vegetables, tomato, garlic, onion, dairy, legumes, and tea decreased.

**Table 1:** Characteristics of HNC patients and controls participated in the IROPICAN study in Iran (2018 and 2020)

**Characteristics**	**Patients**	**Controls**	***P*** ** value**	**Tertiles of DII in patients**				**Tertiles of DII in controls**			
				**T1**	**T2**	**T3**	***P *** **value**	**T1**	**T2**	**T3**	***P *** **value**
Total	876	3409		262	316	298		1138	1136	1135	
Gender			0.001				0.438				0.024
Male	662	2356		201	231	230		760	779	817	
Female	214	1053		61	85	68		378	357	318	
Socio economic status			0.001				0.964				0.420
Poor	342	953		101	120	121		297	336	320	
Moderate	290	1141		89	105	96		389	379	373	
Well	244	1315		72	91	81		452	421	442	
Opium use			0.001				0.394				0.090
No	474	2954		143	162	474		1006	970	978	
Yes	402	455		119	154	129		132	166	157	
Tobacco use			0.001				0.208				0.202
No	340	2305		90	129	121		792	761	752	
Yes	536	1104		172	187	177		346	375	383	
Alcohol use			0.001				0.436				0.433
No	805	3268		237	295	273		1098	1085	1085	
Yes	71	141		25	21	25		40	51	50	
Physical activity			0.694				0.213				0.122
Sedentary	267	1104		85	100	82		352	367	385	
Moderate	200	746		64	62	74		252	236	258	
Heavy	202	749		58	67	77		238	256	255	
Unknown	207	810		55	87	65		296	277	237	
DMFT ^a^			0.001				0.206				0.272
Poor	207	1551		59	75	73		547	511	493	
Moderate	75	449		21	20	34		141	151	157	
Good	594	1409		182	221	191		450	474	485	

*Note.* HNC: Head and neck cancer; DII: Dietary inflammatory index; DMFT: Decayed, missing, or filled teeth. ^a^ Dental health was defined by the DMFT score sum of the number of decayed, missing, or filled teeth.

**Table 2:** Energy-adjusted dietary intakes of participants across case (n = 876) and control (n = 3409) groups and tertiles of the dietary inflammatory index (DII) in the IROPICAN study between 2018 and 2020

						**Tertiles of DII in patients**							**Tertiles of DII in controls**						
	**Patients**		**Controls**			**T1 (n=262)**		**T2 (n=316)**		**T3 (n=298)**			**T1 (n=1138)**		**T2 (n=1136)**		**T3 (n=1135)**		
**Dietary intake**	**Mean**	**SD**	**Mean**	**SD**	***P *** **value**	**Mean**	**SD**	**Mean**	**SD**	**Mean**	**SD**	***P *** **value**	**Mean**	**SD**	**Mean**	**SD**	**Mean**	**SD**	***P *** **value**
DII	1.9	0.8	2.0	0.8	0.173	-2.3	1.0	0.0	0.6	2.5	1.2	0.001	-2.4	1.1	0.0	0.5	2.4	1.1	0.001
Energy	1845.9	796.6	1850.6	682.9	0.860	1780.7	714.4	1648.5	757.6	2112.6	834.1	0.001	1729.5	628.0	1740.9	660.3	2081.9	699.5	0.001
Carbohydrate	302.1	150.7	302.2	127.5	0.971	282.8	128.3	267.2	141.6	355.9	163.2	0.001	274.6	114.2	281.4	118.4	350.8	134.7	0.001
Total protein	65.5	27.3	65.8	24.3	0.817	68.9	28.9	59.5	25.1	68.9	27.4	0.001	66.3	23.0	62.7	24.9	68.3	24.9	0.001
Total fat	45.9	22.0	46.6	20.8	0.464	47.2	20.4	41.6	20.7	49.6	23.9	0.001	46.5	18.5	44.6	21.8	48.6	21.7	0.001
Cholesterol	222.4	147.3	215.3	141.0	0.189	247.9	160.7	201.2	128.4	222.4	150.7	0.001	227.3	136.9	206.7	140.9	211.8	144.5	0.001
SFA	14.8	6.6	14.8	7.3	0.990	14.9	6.6	13.5	6.6	15.9	8.3	0.001	14.6	5.9	14.3	6.9	15.4	6.9	0.001
Trans fatty acids	0.4	0.3	0.4	0.3	0.069	0.4	0.3	0.4	0.2	0.5	0.3	0.001	0.4	0.2	0.4	0.3	0.5	0.3	0.001
MUFA	14.6	6.8	14.8	6.7	0.484	15.1	6.4	13.3	6.5	15.6	7.3	0.001	14.9	5.9	14.1	6.7	15.5	7.1	0.001
PUFA	11.9	7.0	11.9	7.0	0.165	12.3	6.5	10.7	6.7	12.9	7.7	0.001	12.4	6.2	11.6	7.5	12.9	7.3	0.001
Omega-3 fatty acids	1.5	0.9	1.5	0.8	0.213	1.6	0.9	1.3	0.8	1.5	0.9	0.001	1.6	0.7	1.4	0.8	1.5	0.7	0.001
Omega-6 fatty acids	0.1	0.1	0.1	0.1	0.264	0.2	0.1	0.1	0.1	0.1	0.1	0.001	0.1	0.1	0.1	0.1	0.1	0.1	0.002
Fiber	16.9	9.0	17.5	8.0	0.064	19.5	9.3	14.8	7.9	17.0	9.3	0.001	19.5	8.5	16.3	7.8	16.8	7.4	0.001
Fruits	357.5	269.9	362.7	285.7	0.630	346.9	234.6	363.9	287.7	360.1	279.8	0.073	371.3	287.2	371.7	296.7	344.9	271.9	0.037
Vegetables	439.8	407.7	422.8	243.6	0.116	481.2	531.7	411.2	211.2	433.8	437.8	0.115	441.7	247.2	419.4	241.3	407.4	241.2	0.003
Tomato	121.1	175.4	113.6	102.4	0.103	130.9	243.6	113.1	89.5	120.8	170.8	0.486	118.1	105.4	112.2	100	110.5	102.1	0.181
Garlic	1.3	1.6	1.5	2.7	0.062	1.6	1.7	1.2	1.4	1.2	1.7	0.022	1.7	2.8	1.5	3.1	1.2	1.9	0.001
Onion	136.6	190.1	130.1	102.3	0.168	146.9	108.5	128.7	89.4	136.0	296.3	0.521	140.3	115.3	128.9	95.4	120.9	93.9	0.001
Dairy	268.4	181.9	270.5	183.8	0.757	288.9	177.4	270.1	182.3	248.4	184.1	0.031	278.9	212.8	271.8	171.8	260.8	162.6	0.059
Whole grain	39.5	65.4	39.2	74.7	0.914	40.8	64.2	38.9	66.6	39.1	65.6	0.935	38.6	79.3	41.8	77.0	37.3	67.2	0.351
Refined grain	461.4	249.6	453.9	261.5	0.439	467.9	229.4	455.9	230.7	461.6	284.1	0.847	465.5	272.3	442.5	249.9	453.5	261.4	0.112
Sweets	103.9	141.0	102.9	128.6	0.824	113.2	192.5	101.2	111.5	98.8	112.9	0.440	112.3	150.0	100.5	112.8	95.8	119.3	0.007
Total meat	66.9	47.8	68.7	50.8	0.338	67.0	43.4	69.3	46.4	64.2	52.8	0.421	70.2	47.8	66.0	45.8	69.9	57.9	0.088
Red meat	22.2	24.2	23.6	28.3	0.182	24.8	24.6	20.8	20.8	21.2	26.8	0.097	23.6	24.7	23.3	26.5	23.7	33.1	0.925
Processed meat	7.6	14.2	8.9	19.7	0.069	7.5	13.9	8.1	15.2	7.1	13.2	0.668	9.7	21.3	7.8	14.9	9.2	22.0	0.053
Nuts	5.9	7.6	5.9	9.3	0.961	6.6	8.3	5.0	5.3	6.2	8.9	0.026	6.0	10.2	6.0	10.3	5.6	7.2	0.516
Legumes	31.6	39.8	32.1	27.5	0.639	32.4	58.9	31.9	25.6	30.5	30.1	0.829	33.6	27.9	32.2	28.3	30.7	26.1	0.041
Coffee	39.6	94.7	38.3	90.1	0.802	44.4	94.8	41.8	98.1	32.6	91.4	0.549	37.6	83.6	43.4	103.7	34.3	81.5	0.258
Tea	954.4	873.2	1033.2	906.2	0.022	984.9	983.9	1025.8	952.8	853.9	651.0	0.044	1137.9	1030.1	1020.9	856.9	939.2	803.9	0.001

*Note.* DII: Dietary inflammatory index; SD: Standard deviation; SFA: Saturated fatty acids;MUFA: Mono unsaturated fatty acids;PUFA: Poly unsaturated fatty acids. Energy intake is expressed as kcal/day, cholesterol as mg/d, coffee and tea as cc/d, and others are presented as gram/day. Sweets include ice cream, chocolate, cube sugar, sugar, jam, Halva, biscuits, wafers, cookies, carbonated beverages, non-alcoholic beer, and sweetened beverages.

Table 3 presents the association between DII scores and the risk of all subtypes of HNC and total HNC. After adjusting for confounders, a more pro-inflammatory diet was associated with an increased risk of all HNC (OR [95% CI]: 1.31 [1.06-1.62]; *P*-trend = 0.013). Additionally, there was a significant association between DII and lip and oral cavity cancer (OR [95% CI]: 1.56 [1.16-1.66]; *P*-trend = 0.004) in the adjusted model. Notably, an inflammatory diet was associated with an increased risk of pharynx cancer even after adjustment for confounders (OR T3 vs. T1: 2.08 [1.14-3.79]; *P*-trend = 0.02). There was no significant association between DII and larynx cancer, and no interaction was seen between DII and opium use. However, an interaction was found between tobacco use and DII on the risk of HNC, meaning that participants who used tobacco and had higher DII scores were at a greater risk of HNC compared to participants who did not use tobacco and were in the first tertile (OR T3 vs. T1 [95% CI]: 2.52 [1.78-3.57]; *P*-interaction = 0.03, as depicted in Table 4). It is worth noting that participants who did not use tobacco or opium but were in the third tertile of DII were still at great risk of HNC (OR T3 vs. T1 [95% CI]: 1.66 [1.20-2.29]).

**Table 3:** Odds Ratios and Confidence Intervals for the Association Between DII as Continuous Variable Across Tertiles and HNC by Subtypes in the IROPICAN Study of Iran (2018 and 2020)

**Variables**	**T1 **	**T2 **	**T3 **	***P***-trend^*^	**OR (95% CI)**
All HNC					
Case/control	262/1138	316/1136	298/1135	-	-
Crude	Ref.	1.2 (1.01, 1.45)	1.14 (0.95, 1.37)	0.173	1.04 (1.00, 1.07)
Adjusted^*^	Ref.	1.13 (0.92, 1.39)	1.31 (1.06, 1.62)	0.013	1.07 (1.03, 1.12)
Lip and oral cavity					
Case/control	88/1138	116/1136	109/1135	-	-
Crude	Ref.	1.3 (0.99, 1.76)	1.24 (0.93, 1.66)	0.16	1.05 (0.99, 1.11)
Adjusted	Ref.	1.33 (0.99, 1.79)	1.56 (1.16, 1.66)	0.004	1.12 (1.05, 1.19)
Pharynx					
Case/Control	19/1138	28/1136	35/1135	-	-
Crude	Ref.	1.48 (0.82, 2.66)	1.85 (1.05, 3.25)	0.03	1.09 (0.99, 1.22)
Adjusted	Ref.	1.49 (0.82, 2.72)	2.08 (1.14, 3.79)	0.02	1.14 (1.02, 1.28)
Larynx					
Case/control	139/1138	153/1136	135/1135	-	-
Crude	Ref.	1.10 (0.86, 1.41)	0.97 (0.76, 1.25)	0.84	1.01 (0.97, 1.06)
Adjusted	Ref.	0.88 (0.65, 1.21)	0.89 (0.65, 1.24)	0.50	1.01 (0.95, 1.07)

*Note.* DII: Dietary inflammatory index; HNC: Head and neck cancer; OR: Odds ratio; CI: Confidence interval; Adjusted for energy (kcal/d), age (years), gender (female, male), province (10 provinces), social economic status (low, medium, and high), tobacco use (yes, no), opium use (yes, no), alcohol use (yes, no), dental health (poor, moderate, and good), and physical activity (sedentary, moderate, and high). The difference in the association between DII and the risk of subtypes of HNC is significant (*P* of heterogeneity = 0.03).

**Table 4:** Odds Ratios of HNC Stratified by Opium and Tobacco in Relation to DII Treated as Continuous Variable

**Variables**	**T1**		**T2**		** T3**		
	**Case/Control**	**OR (95% CI)**	**Case/Control**	**OR (95% CI)**	**Case/Control**	**OR (95% CI)**	***P***_interaction_
Cigarette smoking							0.264
No	110/844	Ref.	139/818	1.29 (0.97, 1.70)	133/786	1.5 (1.13, 2.01)	
Yes	152/294	2.34 (1.69, 3.24)	171/318	2.27 (1.65, 3.14)	165/349	2.57 (1.86, 3.56)	
Water pipe use							0.271
No	227/1056	Ref.	293/1054	1.19 (0.96, 1.47)	276/1075	1.32 (1.05, 1.65)	
Yes	35/82	1.33 (0.83, 2.14)	23/82	0.91 (0.54, 1.55)	22/60	1.52 (0.91, 2.78)	
Opium use							0.120
No	79/759	Ref.	112/723	1.54 (1.13, 2.11)	105/710	1.78 (1.28, 2.46)	
Yes	119/132	4.8 (2.89, 7.97)	154/166	4.67 (2.85, 7.68)	129/157	5.57 (3.35, 9.26)	

*Note.* HNC: Head and neck cancer; DII: Dietary inflammatory index; OR: Odds ratio; CI: Confidence interval.

## Discussion

 This study found that DII is positively associated with HNC risk overall. In addition, there was a significant association between DII and lip and oral cavity cancer and pharynx cancer. However, no significant association was found between DII and larynx cancer. There was an interaction between tobacco use and DII in determining the risk of HNC, whereas no interaction effect was found between opium use and DII.

 DII is a marker of the pro-inflammatory potential of the diet. Some studies with large sample sizes investigated the association between DII and HNC worldwide, but they are limited, especially in low and middle-income countries.^12,15,16,23,24^ Our overall findings are consistent with these studies, indicating that a pro-inflammatory diet, as indicated by higher DII scores, is associated with HNC.

 Previous studies evaluated the interaction effect of tobacco or smoking with DII on HNC risk, however, but none of them have assessed the interaction between opium use and DII on HNC.^16^ Shivappa et al reported a positive association between DII and laryngeal cancer in a case-control study in Italy,^25^ which is in contrast with our findings. Furthermore, we found a positive significant association between DII and pharynx cancer which is in line with Shivappa’s case-control study in Italy.^23^ Mazul et al reported a significant interaction between smoking, alcohol intake, and DII on HNC risk.^16^ We assessed opium use in detail, including its interaction effect, finding no interaction effect between opium use and DII on HNC risk. Overall, tobacco use was more prevalent in our population compared to opium use. Moreover, based on the information in Table 1, opium use was not consistent with DII tertiles, indicating that the distribution of opium users in the DII tertiles is not similar. Therefore, we cannot conclude that opium users have a more pro-inflammatory diet.

 Evidence suggested that the intake of a diet high in pro-inflammatory parameters accompanied by smoking could exacerbate carcinogenesis. Smoking and alcoholic consumption have been linked with increased oxidative stress.^26,27^ Smoking-associated oxidative stress activates the inflammatory response pathway, triggering a cascade of events in which the production and release of reactive oxygen species (ROS) at the site of damage and inflammation potentially increase oxidative damage to macromolecular targets, which may lead to cancer initiation and progression.^28^

 The positive association between the DII and HNC might be mediated through the excess production of cytokines such as interleukin-6 (IL-6), IL-8, platelet-derived growth factor, and vascular endothelial growth factor in the tumor microenvironment, which are responsible for carcinogenic activities like anti-apoptosis, tumor angiogenesis, and metastasis.^29^ Inflammatory cytokines can affect the oral microbiota, which in turn can cause an increased risk of periodontitis and cancer.^30-32^ Moreover, the direct impact of carbohydrate consumption on the mouth microbiota, leading to increased populations of lactobacilli bacteria, can be another pathway.^3^ The oral cavity, pharynx, and larynx are all connected anatomical sites, so dysbiosis in one site can lead to dysbiosis in another.^34^

 This study has several strengths. To our knowledge, this is the first large multicenter case-control study in the East Mediterranean region examining the association between DII and the risk of HNC. Additionally, the large sample size allowed us to study the association between DII and overall HNC and its subsites, including oral cavity and larynx cancers. The patients were pathologically confirmed by a pathologist. We also adjusted for several confounding variables, including tobacco, opium, and alcohol use, province, socioeconomic status, dental health, and physical activity. Using a validated FFQ was another strength of the current study.^20^

 However, this study suffers from some limitations. The lack of an Iranian food composition table is one of the main limitations as food components would differ by country due to environmental and food processing factors. However, using the international food composition table minimizes this problem. Moreover, several Iranian studies showed an association between DII scores calculated using this food composition table, inflammatory index, and health status.^35-37^

**Highlights**

- The inflammatory potential of diet may affect carcinogenesis.
- In this multicenter case-control study, we assessed dietary inflammatory index (DII) of 876 new cases of head and neck cancer (HNC) and 3409 healthy controls.
- DII was positively associated with HNC risk.
- There was an interaction between tobacco use and DII in determining the risk of HNC.

## Conclusion

 In conclusion, subjects who consumed a pro-inflammatory diet had an increased risk of lip and oral cavity cancer and pharynx cancer compared to those who consumed a low-inflammatory diet in this Iranian population. This is the first study in Iran to examine this association, and the results suggest that increasing the intake of anti-inflammatory dietary factors such as plant-based foods rich in fiber and phytochemicals and reducing the intake of pro-inflammatory factors such as fried or processed foods rich in saturated fat or animal protein may be a strategy for reducing the risk of HNC. This can also be the key measure of avoiding tobacco use, indicating that the interaction between a pro-inflammatory diet and tobacco use leads to strikingly higher risks of HNC.

## Acknowledgments

 This study was conducted using data from the IROPICAN study, which was funded by the National Institute for Medical Research Development (NIMAD) with project No.17198. The authors thank all participants and the healthcare workers who patiently support this study.

## Authors’ Contribution

**Conceptualization**: Kazem Zendehdel, Fatemeh Toorang, Saba Narmcheshm.

**Data curation:** Fatemeh Toorang, Saba Narmcheshm.

**Formal analysis:** Fatemeh Toorang, Saba Narmcheshm, Bahareh Sasanfar.

**Funding acquisition:** Kazem Zendehdel, Paolo Boffetta.

**Investigation:** Kazem Zendehdel, Fatemeh Toorang, Monireh Sadat Seyyedsalehi, Elham Mohebbi, Ahmad Naghibzadeh-Tahami, Hamideh Rashidian, Bahareh Sasanfar.

**Methodology**: Kazem Zendehdel, Fatemeh Toorang, Paolo Boffetta, Saba Narmcheshm, Elham Mohebbi, Maryam Hadji.

**Project administration:** Fatemeh Toorang, Monireh Sadat Seyyedsalehi, Elham Mohebbi, Ahmad Naghibzadeh-Tahami, Hamideh Rashidian, Maryam Hadji.

**Resources:** Paolo Boffetta, Kazem Zendehdel.

**Software:** Fatemeh Toorang, Bahareh Sasanfar.

**Supervision:** Kazem Zendehdel, Fatemeh Toorang.

**Validation:** Fatemeh Toorang, Saba Narmcheshm.

**Visualization:** Kazem Zendehdel, Fatemeh Toorang, Paolo Boffetta, Saba Narmcheshm.

**Writing–original draft: **Saba Narmcheshm.

**Writing–review & editing**: Saba Narmcheshm, Kazem Zendehdel, Fatemeh Toorang, Paolo Buffetta.

## Competing Interests

 The authors declare no potential conflict of interests.

## Ethical Approval

 The IROPICAN study received the ethical code from the NIMAD committee (IR.NIMAD.REC.1394.027). None of the data are published in the name of the participants.

## Funding

 This study analyzed the IROPICAN study, which was granted by NIMAD (project code: No.17198).It was partially supported by a grant from the Italian Association for Cancer Research (IACR) (No. GI 60742).
